# Supplementary material for: Screening the Coulomb interaction leads to a prethermal regime in two-dimensional bad conductors
Source: Nat Commun. 2023 Nov 2;14:7004. doi: 10.1038/s41467-023-42778-2 (PMC10622453; doi:10.1038/s41467-023-42778-2)
Supplement: Supplementary file 1 — Supplementary Information [file 41467_2023_42778_MOESM1_ESM.pdf]

Supplementary Information for

Screening the Coulomb interaction leads to a prethermal regime in two-dimensional bad conductors

L. J. Stanley[1,2], Ping V. Lin[1,3], J. Jaroszyński[1], Dragana Popović[1,2*]

[1]National High Magnetic Field Laboratory, Florida State University,
Tallahassee, Florida 32310, USA
[2]Department of Physics, Florida State University,
Tallahassee, Florida 32306, USA
[3] Department of Physics, Zhejiang Sci-Tech University,
Hangzhou 310018, China
[*]To whom correspondence should be addressed; E-mail: dragana@magnet.fsu.edu

Supplementary Figures

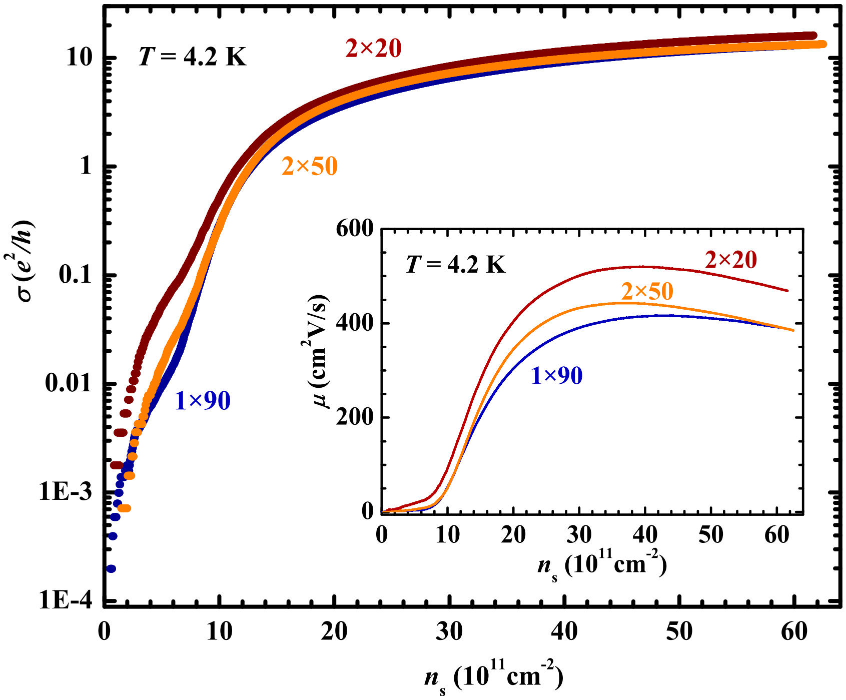

Supplementary Fig. 1: **Dependence of the conductivity σ on carrier density n_s in thin-oxide devices at $T = 4.2$ K.** The data are shown for three representative samples with dimensions $L \times W$ (L - length, W - width) of 2×20, 2×50, and 1×90 $\mu\mathrm{m}^2$. Measurements were performed with the samples immersed in liquid helium. Inset: Mobility $\mu = \sigma/en_s$ vs n_s, corresponding to the data in the main figure. The relatively low values of the peak mobility at $T = 4.2$ K reflect the presence of a large amount of disorder. Similar results were obtained in thick-oxide devices[1–5].

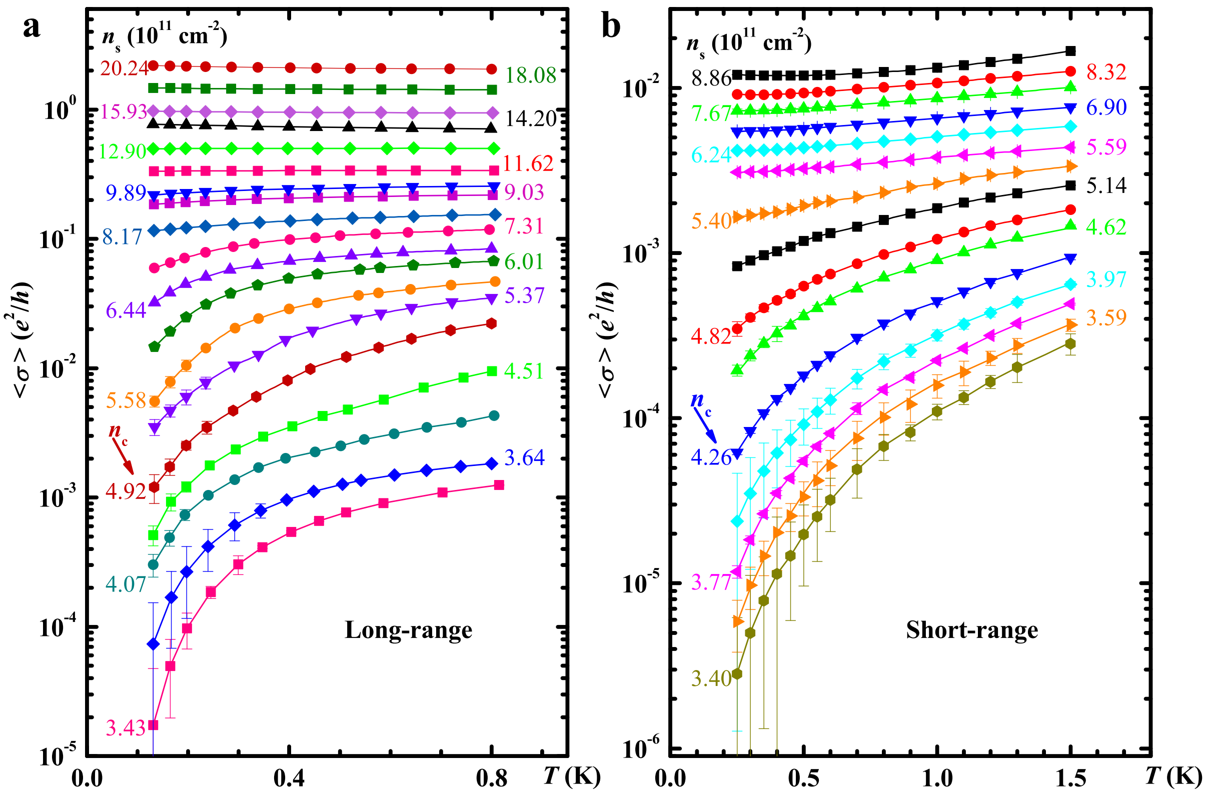

Supplementary Fig. 2: **Comparison of the dc transport in 2DESs with the long-range and screened Coulomb interactions.** Conductivity $\langle\sigma\rangle$ vs T for different n_s, as shown, for the case of **a** long-range Coulomb interaction (adapted from ref.[1]) and **b** screened Coulomb interaction (adapted from ref.[6]). n_s was varied at high $T \approx 20$ K, followed by cooling to a desired T. σ was measured as a function of time, up to several hours at the lowest n_s and T. $\langle\ldots\rangle$ indicates averaging over time, with the error bars corresponding to 1 S.D. of the fluctuations with time. The dimensions of both samples were 1×90 μm^2. The critical densities n_c for the metal-insulator transition are marked by arrows.

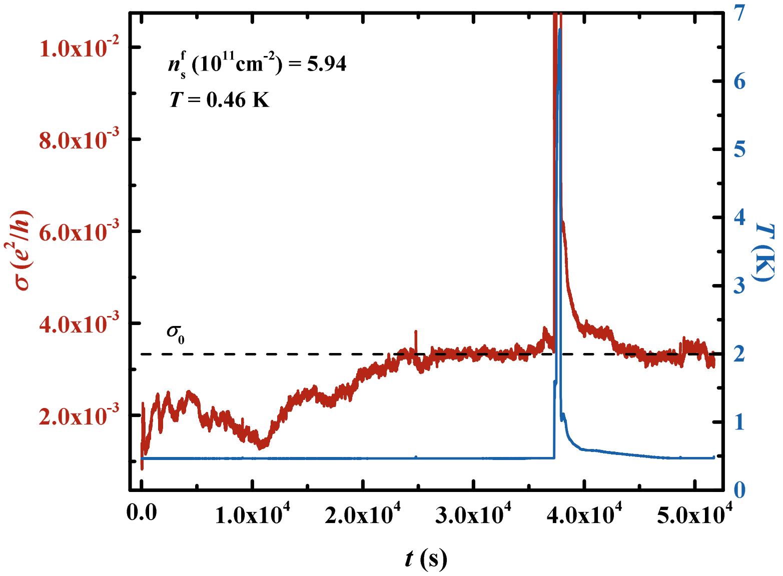

Supplementary Fig. 3: Stationary state conductivity σ_0 achieved after a quench from higher density and after cooling from high temperature (T). Short-range case; sample 2 × 50. After σ_0 (dashed line) is reached following a change of density from $n_s^i(10^{11}\text{cm}^{-2}) = 32.5$ to $n_s^f(10^{11}\text{cm}^{-2}) = 5.94$ at $T = 0.46$ K, the temperature is raised to ~ 7 K, and then the sample is cooled back to $T = 0.46$ K with $n_s = n_s^f(10^{11}\text{cm}^{-2}) = 5.94$. The same σ_0 is obtained in both cases.

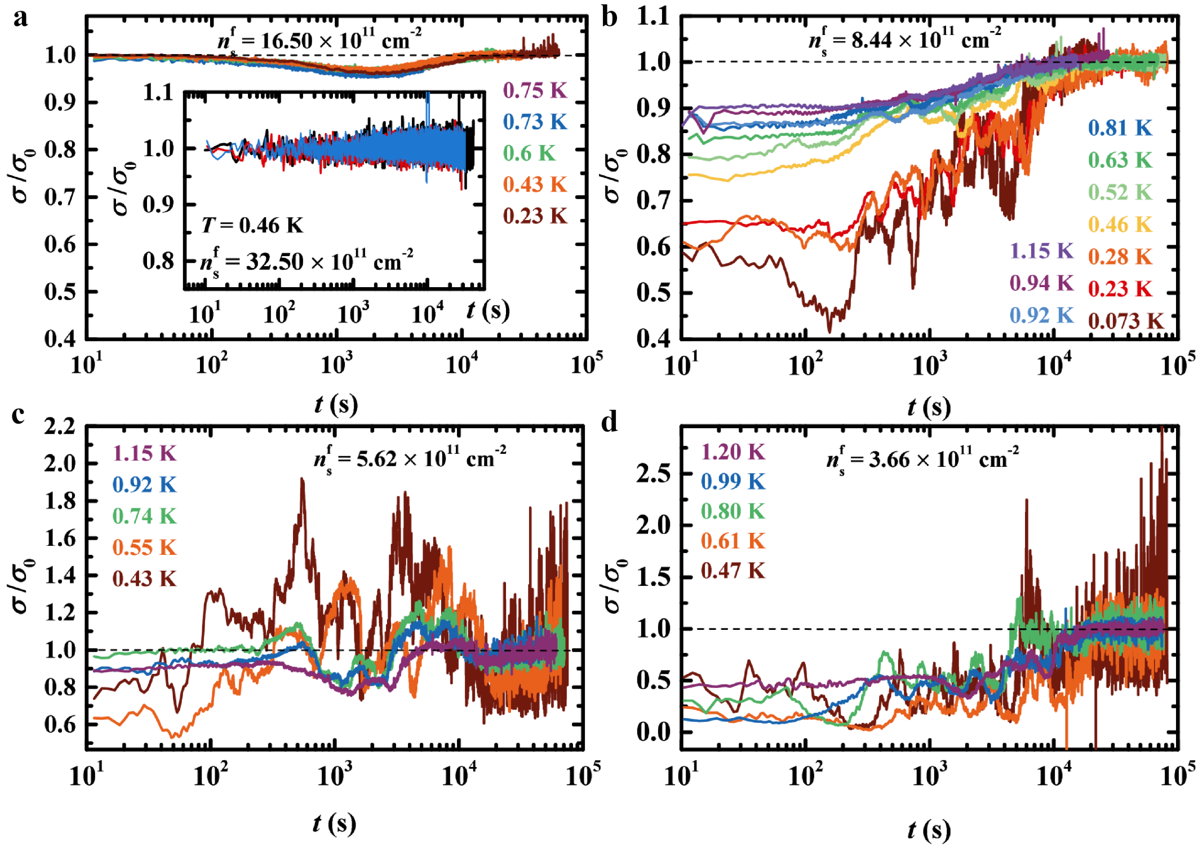

Supplementary Fig. 4: Relaxations of σ normalized by the stationary value $\sigma_0(n_s^f, T)$ for different final densities and T; short-range case. Sample 2×20 and $n_s^i(10^{11}\text{cm}^{-2})= 32.20$, unless noted otherwise. **a** $n_s^f(10^{11}\text{cm}^{-2})= 16.50$ and different T, as shown; $\sigma_0(T) \sim e^2/h$. Inset: Sample 2×50; $n_s^f(10^{11}\text{cm}^{-2})= 32.50$ with $n_s^i(10^{11}\text{cm}^{-2})=$ 3.97 (black), 5.94 (red), and 8.75 (blue) at $T = 0.46$ K. Here n_s^f is high enough such that $\sigma_0 > e^2/h$, and no relaxations are observed regardless of the value of n_s^i. **b** $n_s^f(10^{11}\text{cm}^{-2})=$ 8.44 and different T, as shown. **c** $n_s^f(10^{11}\text{cm}^{-2})= 5.62$ and different T, as shown. **d** $n_s^f(10^{11}\text{cm}^{-2})= 3.66 < n_c$ and different T, as shown. Horizontal dashed lines mark $\sigma = \sigma_0(n_s^f, T)$.

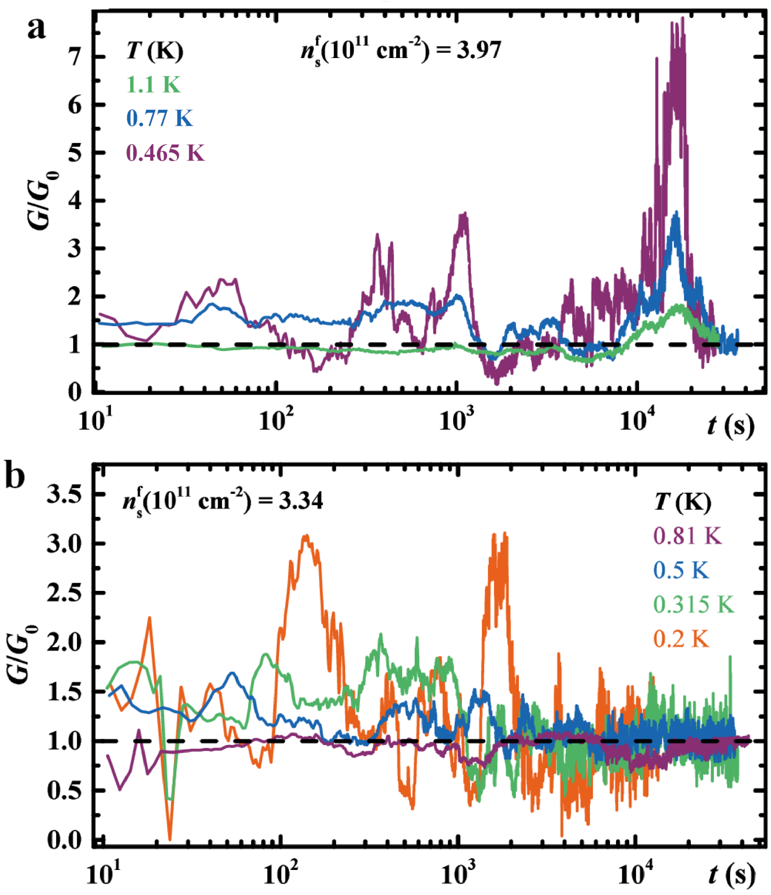

Supplementary Fig. 5: **Relaxations of σ normalized by the stationary value $\sigma_0(n_s^f, T)$ for final densities in the insulating regime; short-range case. a** Sample 2×50; $n_s^i(10^{11}\ \mathrm{cm}^{-2})$= 32.50, $n_s^f(10^{11}\ \mathrm{cm}^{-2})$= 3.97, and different T, as shown. **b** Sample 1×90; $n_s^i(10^{11}\ \mathrm{cm}^{-2})$= 31.90, $n_s^f(10^{11}\ \mathrm{cm}^{-2})$= 3.34, and different T, as shown. Horizontal dashed lines mark $\sigma = \sigma_0(n_s^f, T)$.

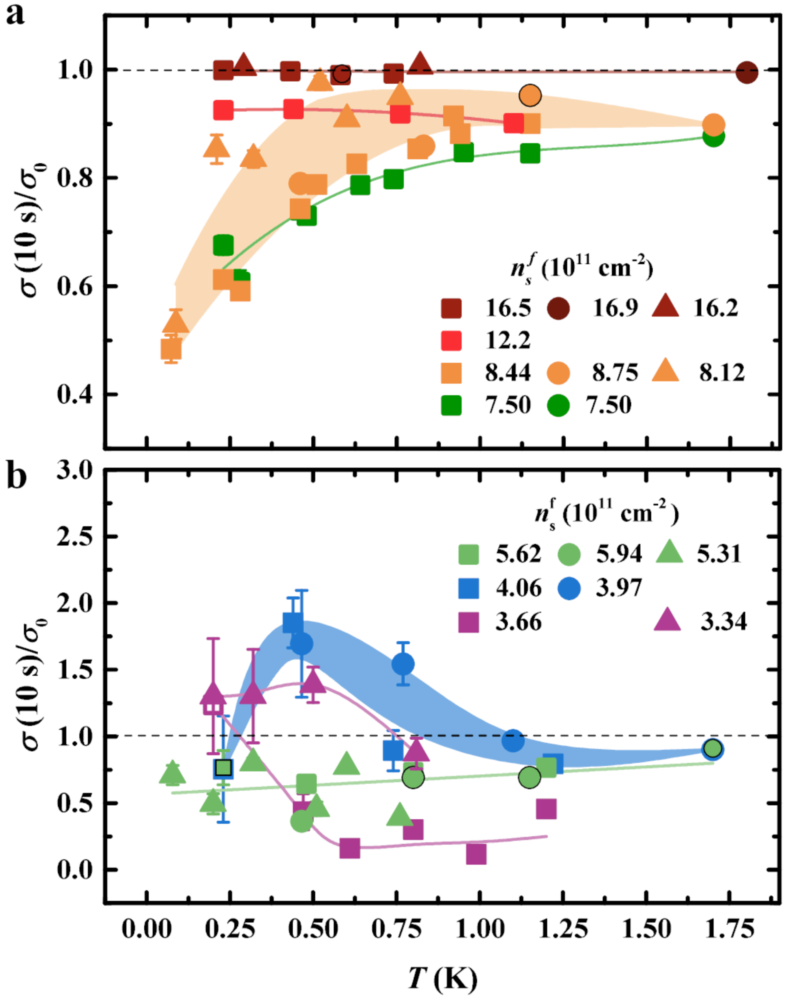

Supplementary Fig. 6: **Temperature dependence of the initial $\boldsymbol{\sigma}(10\ \mathrm{s})/\boldsymbol{\sigma}_0$.** Temperature dependence of $\sigma(10\ \mathrm{s})/\sigma_0$ for **a** $n_{\mathrm{s}}^{\mathrm{f}}$ well above n_{c} and **b** $n_{\mathrm{s}}^{\mathrm{f}}$ around n_{c}. For all panels the symbol shape indicates both the size of the sample and the initial density $n_{\mathrm{s}}^{\mathrm{i}}$ (10^{11} cm^{-2}); the latter is 32.2 for the square (sample 2×20), 32.2 for the circle (sample 2×50), and 31.9 for the triangle (1×90). Solid lines and shaded regions are guides for the eye. Dashed black lines indicate the apparent equilibrium value; open symbols describe the data obtained on another sample with the same dimensions. The error bars reflect 1 S.D. of the fluctuations of σ_0 with time. As shown in **a**, at very large $n_{\mathrm{s}}^{\mathrm{f}}$, $\sigma/\sigma_0 \approx 1$ and it does not depend on T. As $n_{\mathrm{s}}^{\mathrm{f}}$ is reduced within the metallic regime (albeit $k_{\mathrm{F}}l < 1$), the deviations of σ/σ_0 from 1 increase smoothly with decreasing T. However, as $n_{\mathrm{s}}^{\mathrm{f}} \to n_{\mathrm{c}}(10^{11}\mathrm{cm}^{-2}) = (4.2 \pm 0.2)$, the behavior drastically changes (see **b**), consistent with the presence of a phase transition. In addition, in the insulating regime ($n_{\mathrm{s}}^{\mathrm{f}} < n_{\mathrm{c}}$) where relaxations are dominated by the noise (Supplementary Figs. 4d and 5), the initial amplitudes $\sigma(10\ \mathrm{s})/\sigma_0$ become sample dependent.

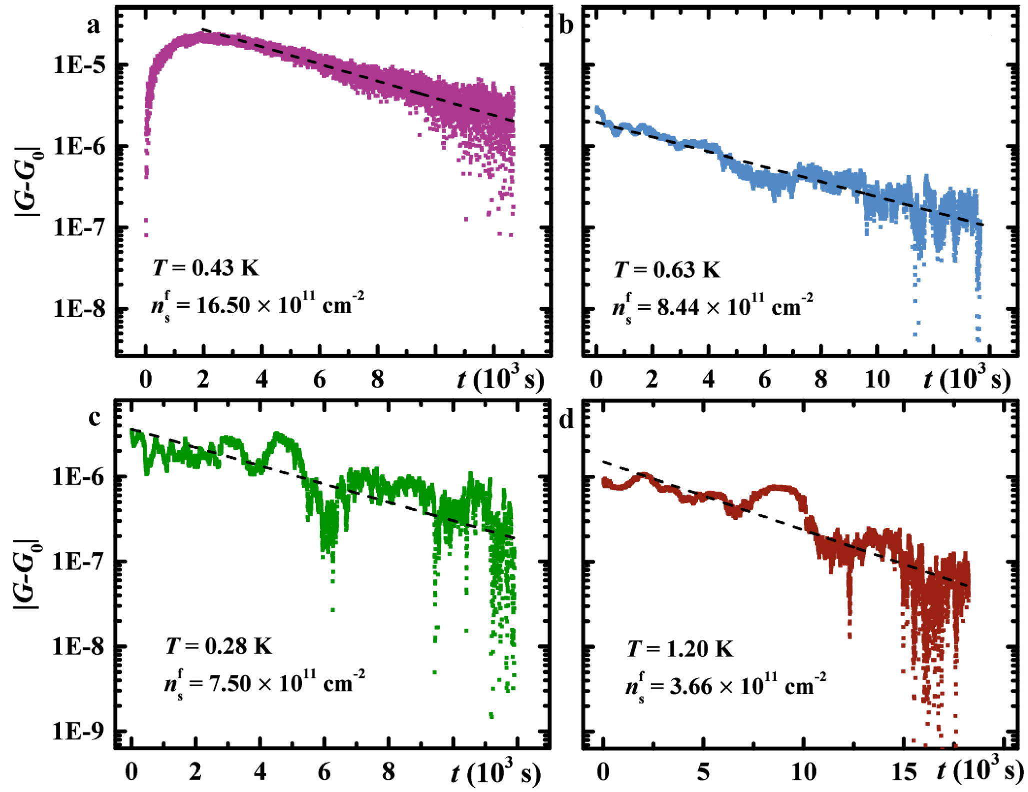

Supplementary Fig. 7: **Exponential relaxations of the conductance G in the short-range case with sample in vacuum.** Black dashed lines are fits to $|G - G_0| \propto \exp[-(t/\tau_\sigma)]$, where τ_σ is the characteristic time to reach the apparent equilibrium conductance G_0, for n_s^f and T, as shown; $n_s^i(10^{11}\ \mathrm{cm}^{-2}) = 32.20$; sample 2×20. **a** $\tau_\sigma = (9470 \pm 50)$ s; the same data are shown also in Supplementary Fig. 4a. **b** $\tau_\sigma = (10880 \pm 50)$ s; the same data are shown also in Supplementary Fig. 4b. **c** $\tau_\sigma = (9280 \pm 80)$ s; the same data are shown also in Fig. 2b. **d** $\tau_\sigma = (12440 \pm 80)$ s; the same data are shown also in Supplementary Fig. 4d.

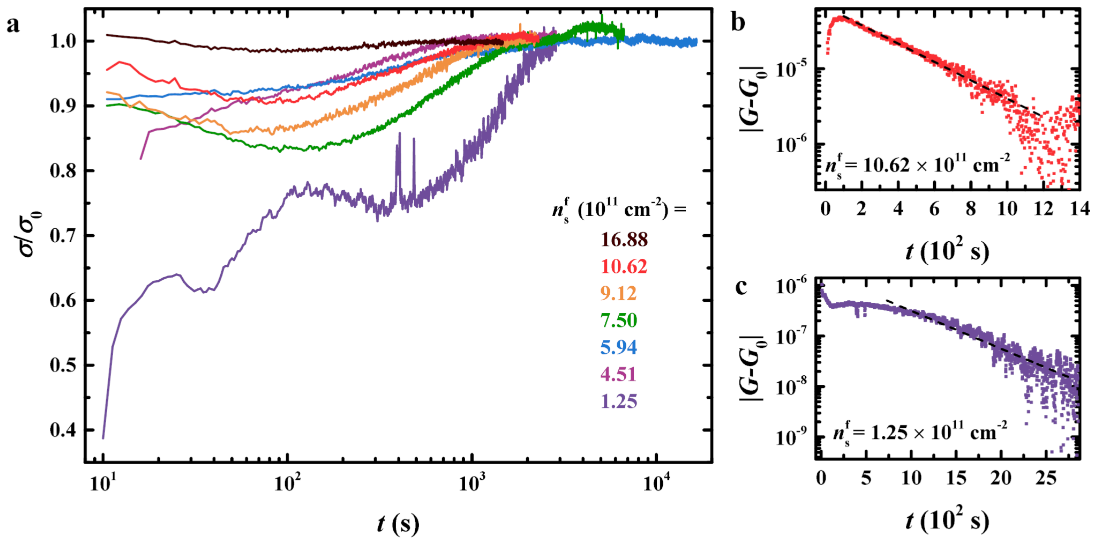

Supplementary Fig. 8: **Relaxations in the short-range case with sample in ^{4}He vapor.** **a** Relaxations of σ normalized by the apparent equilibrium value $\sigma_0(n_s^f, T)$ for several n_s^f, as shown; $T = 1.7$ K, sample 2×50. **b** and **c** show exponential fits (black dashed lines) to $|G - G_0| \propto \exp[-(t/\tau_\sigma)]$ at long times (G is the conductance) for two different n_s^f, as shown; $\tau_\sigma = (820 \pm 7)$ s and $\tau_\sigma = (2200 \pm 200)$ s in **b** and **c**, respectively.

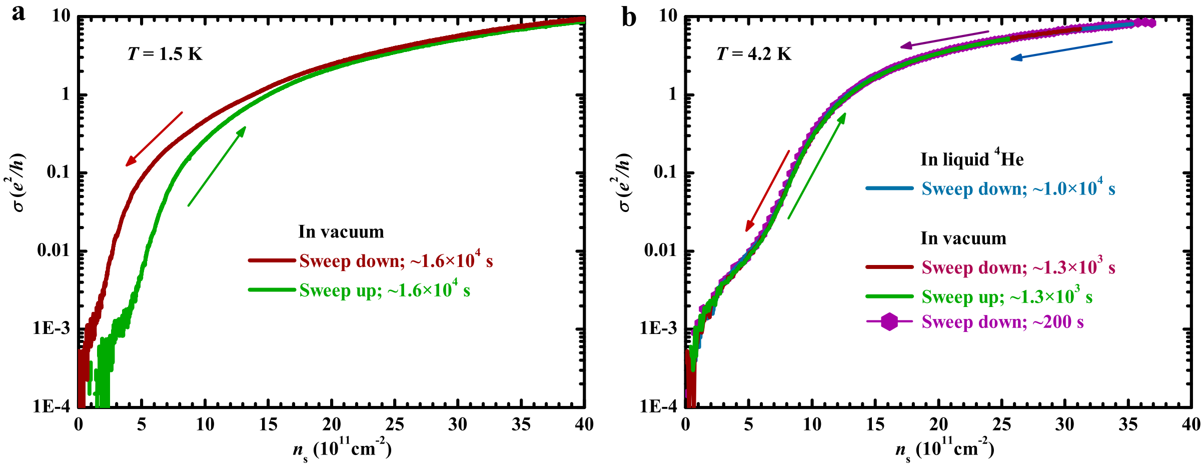

Supplementary Fig. 9: **Conductivity $\sigma(n_s)$ for different n_s sweep rates.** Short-range case; sample 1×90. **a** $T = 1.5$ K; sample in vacuum. The hysteresis in $\sigma(n_s)$ observed for a sweep $\sim 1.6 \times 10^4$ s long, implies that τ_σ must be even longer. **b** $T = 4.2$ K; sample measured in both vacuum and liquid helium. The absence of a hysteresis and the same values of $\sigma(n_s)$ obtained in both set-ups and regardless of the sweep rate, indicate that τ_σ must be lower than ~ 200 s and that thermalization is dominated by electron-phonon coupling between the 2DES and bulk Si.

Supplementary Note 1

Metal-insulator transition

Figure S2 shows the time-averaged $\langle\sigma\rangle$ as a function of T for different n_s for both sets of samples (see also refs.[1,6]). The critical densities n_c for the metal-insulator transition were determined[1,6] from the fits to $\langle\sigma(n_\mathrm{s},T)\rangle$ on both metallic and insulating sides of the transition, in particular, from i) the vanishing of the activation energy in the exponential $\langle\sigma(T)\rangle$ found in the insulating regime at the lowest n_s, and ii) the extrapolated zero-temperature conductivities $\langle\sigma(n_\mathrm{s},T=0)\rangle > 0$ on the metallic side of the transition. Notably, both methods yield the same values of n_c. At the critical density, a simple power-law T dependence is observed, $\langle\sigma(n_\mathrm{c},T)\rangle \propto T^x$ ($x = 1.5$). This means that $\langle\sigma(n_\mathrm{c},T)\rangle$ belongs to the insulating family of curves for which $\langle\sigma(T=0)\rangle = 0$. Furthermore, it has been shown for both sets of samples[6] that the conductivity near the MIT can by described by a scaling form $\langle\sigma(n_\mathrm{s},T)\rangle = \langle\sigma_\mathrm{c}(T)\rangle f(T/T_0)$, consistent with the existence of a quantum phase transition. A careful scaling analysis further confirms the extrapolations of $\langle\sigma(n_\mathrm{s},T)\rangle$ to $T=0$.

These results on 2DESs in high-disorder Si MOSFETs illustrate that the mere decrease of $\langle\sigma\rangle$ with decreasing T (i.e., $d\langle\sigma\rangle/dT > 0$) at a given n_s does not necessarily imply the existence of an insulating state ($\langle\sigma(T=0)\rangle = 0$). Indeed, the existence of a 2D metal ($\langle\sigma(T=0)\rangle \neq 0$) with $d\langle\sigma\rangle/dT > 0$ has been demonstrated also in low-disorder Si MOSFETs in the spin-polarized regime[7] and in the presence of disorder-induced local magnetic moments[8,9] as well as in layered 2D materials such as ReS_2 (ref.[10]) and WSe_2 (ref.[11]). Of course, it is well-known that similar behavior is found also in conventional semiconductors such as doped silicon, where the 3D metal-insulator transition occurs between a metallic state with $d\sigma/dT > 0$ and $\sigma(T=0) \neq 0$, and an insulating state with $d\sigma/dT > 0$ and $\sigma(T=0) = 0$ (ref.[12]). Therefore, in all those cases the critical density

n_c for the MIT is lower than the density at which $d\sigma/dT = 0$. In 2D, such behavior does not agree with the simplest scaling scenario ("Wegner scaling") according to which the exponent $x = (D - 2)/z = 0$ (z is the dynamical exponent), so the critical conductivity should not depend on T. It is for this reason that many early studies of the 2D MIT (mis)identified n_c as the carrier density at which $d\sigma/dT$ changes sign from insulatorlike ($d\sigma/dT > 0$) to metallic ($d\sigma/dT < 0$). However, it should be emphasized that scaling with $x \neq 0$ for 2D systems does not contradict any fundamental principle and, in fact, it has been predicted for certain microscopic models (see ref.[13] for review and refs. therein). Indeed, it is precisely the general scaling form with $x \neq 0$ that provides a satisfactory and consistent description of all the data near a 2D MIT.

Supplementary Note 2

Intrinsic nonequilibrium behavior of a 2DES

Comprehensive studies of charge dynamics in a *high-disorder* 2DES with a *long-range* Coulomb interaction have demonstrated that the glassy behavior of the electrons is intrinsic rather than their response to extrinsic slow degrees of freedom [e.g. charging of the Si-SiO_2 interface traps or glassiness of the background disorder potential, although exponentially unlikely at our measurement T (ref.[14])]. Here we summarize some of the relevant evidence.

a) The same quantum quench protocol that is discussed in this work, i.e. where n_s is changed rapidly by a large amount ($k_B T \ll E_F < \Delta E_F$), was used to repeat some of the measurements for different values of a back-gate (substrate) bias V_{bg} (ref.[3]). In addition to changing the disorder, it is known that V_{bg} moves the position of the 2D subband with respect to the bottom of the Si conduction band, and it also affects the splitting between the subbands of the 2DES (see ref.[3] and refs. therein). If the exponential process at long

t, which determines $\tau_\sigma \propto \exp(-E_{\rm A}/T)$, where $E_A \approx 57$ K, results from an activation to Si-SiO_2 traps or to an upper subband, it can be estimated that the applied range $-5 \leq V_{\rm bg}$(V)≤ 0 will have a significant impact on $E_{\rm A}$. However, no change in $E_{\rm A}$ was found[3], strongly suggesting that the above two processes can be ruled out as mechanisms for σ to reach a stationary value σ_0.

It should be also noted that the form of the glassy, nonexponential $\sigma(t)$ observed at intermediate times did not depend on the initial density $n_{\rm s}^{\rm i}$, but only on $n_{\rm s}^{\rm f}$ (albeit the overshooting of σ_0 manifests itself as a maximum, not a minimum, in $\sigma(t)$ when $n_{\rm s}^{\rm i} < n_{\rm s}^{\rm f}$) (refs.[2,3]). In other words, the same results were obtained for a given $n_{\rm s}^{\rm f}$ regardless of the direction of the gate-voltage step[3] or step size[4], thus providing further support for the intrinsically glassy nature of the electron dynamics.

b) The waiting-time protocol, in which $\sigma(t)$ was measured after a large, temporary change of $n_{\rm s}$ to another value during the waiting time $t_{\rm w}$, was used to demonstrate aging or the loss of time translation invariance, the key characteristic of relaxing glassy systems[3,4,15].

• An abrupt change in the aging properties was found precisely at the 2D MIT[4,15]. This indicates that, even if the background disorder potential is glassy, the 2DES affects the properties of the background, i.e. that 2D electrons cannot be thought of as simply following the time-dependent changes of the background that is independent of the 2DES, but rather that together they represent a coupled, strongly interacting system.

• The change in the aging properties as a function of $n_{\rm s}$ can be described neither quantitatively nor qualitatively by the dependence of aging on the $V_{\rm g}$ step size[4], which further rules out charging of interface traps as a possible origin of the slow relaxations.

• Importantly, when all the electrons were removed from the 2D layer during $t_{\rm w}$, it was shown[3] that subsequent relaxations did not depend on $t_{\rm w}$. This is in contrast to what

one would expect if the slow dynamics was dominated by the response of the electrons to extrinsic slow degrees of freedom. In that case, $\sigma(t)$ measured after the electrons were reintroduced with the same initial n_s would depend on t_w. Therefore, the experimental findings confirm the existence of glassiness in the 2DES itself.

c) In the case of small perturbations, such that $k_B T < \Delta E_F \ll E_F$, there were no observable relaxations of σ, but large non-Gaussian conductance noise emerged[1,5] for $n_s < n_g$ (n_g is the glass transition density), i.e. in precisely the same regime where relaxations exhibit out-of-equilibrium, glassy behavior. A detailed study of several noise statistics (the probability density function, power spectrum, and the second spectrum, which is a fourth-order noise statistic) supports the conclusions of the relaxation studies.

d) The conductance noise was studied[5] also after cooling with a fixed n_s. This is a small perturbation since $k_B T < k_B \Delta T \ll E_F$ and thus, there were no visible relaxations of σ. This protocol does not involve any change in n_s and, yet, the emergence of non-Gaussian noise for $n_s < n_g$ was also observed, consistent with the above noise and relaxation studies.

e) Finally, as already noted in ref.[3], the characteristic RC charging times of the devices and the circuit are at most 10 ms, so that slow relaxations observed at times longer than a few seconds cannot be attributed to the slow change of the total (average) n_s in the 2D inversion layer.

Conductance noise was studied also in *low-disorder* Si MOSFETs with a *long-range* Coulomb interaction[7,16]. Although those devices had been fabricated in a very different way[17], the results provided the same evidence for glassy freezing of the 2DES below a well-defined $n_s = n_g$ as in high-disorder samples. This strongly suggests the intrinsic nature of the observed glassy dynamics. Moreover, the noise studies on a spin-polarized 2DES indicate that glassy freezing is due to charge, as opposed to spin, degrees of freedom[7].

Since *high-disorder* Si MOSFETs with a short-range and those with a long-range Coulomb interaction were fabricated simultaneously, i.e. under identical conditions, any extrinsic effects (disorder potential, interface traps...) are expected to be comparable. Therefore, the striking, qualitative difference in the dynamical behavior of the 2DESs in these two cases, as illustrated in Fig. 2, presents a clear and convincing evidence for the intrinsic nature of the observed dynamics and the effect of the range of the Coulomb interactions.

Supplementary Note 3

Determination of electron density in Si MOSFETs

For completeness, we mention that in Si MOSFETs at high carrier densities, such that $k_F l > 1$, electron density can be determined also from transport measurements in perpendicular magnetic fields (B). The carrier density obtained from low-temperature Hall measurements at relatively low fields ($\omega_c \tau_s < 1$, where ω_c is the cyclotron frequency and τ_s is the scattering time) is typically[14] somewhat different from $n_s = C_{ox}(V_g - V_{th})/e$. It may also depend on the values of B and T used in the Hall measurement[18] because of the quantum corrections to the conductivity, in particular, electron-electron interactions in the presence of disorder. However, at low carrier densities in the $k_F l < 1$ regime, which is the subject of our study, the Hall effect is not well understood, and thus it remains unclear how to relate Hall measurements to electron density.

Supplementary References

1. Bogdanovich, S. & Popović, D. Onset of glassy dynamics in a two-dimensional electron system in silicon. *Phys. Rev. Lett.* **88**, 236401 (2002).

2. Jaroszyński, J. & Popović, D. Nonexponential relaxations in a two-dimensional electron system in silicon. *Phys. Rev. Lett.* **96**, 037403 (2006).

3. Jaroszyński, J. & Popović, D. Nonequilibrium relaxations and aging effects in a two-dimensional Coulomb glass. *Phys. Rev. Lett.* **99**, 046405 (2007).

4. Jaroszyński, J. & Popović, D. Aging effects across the metal-insulator transition in two dimensions. *Phys. Rev. Lett.* **99**, 216401 (2007).

5. Lin, P. V., Shi, X., Jaroszynski, J. & Popović, D. Conductance noise in an out-of-equilibrium two-dimensional electron system. *Phys. Rev. B* **86**, 155135 (2012).

6. Lin, P. V. & Popović, D. Critical behavior of a strongly disordered 2D electron system: The cases of long-range and screened Coulomb interactions. *Phys. Rev. Lett.* **114**, 166401 (2015).

7. Jaroszyński, J., Popović, D. & Klapwijk, T. M. Magnetic-field dependence of the anomalous noise behavior in a two-dimensional electron system in silicon. *Phys. Rev. Lett.* **92**, 226403 (2004).

8. Feng, X. G., Popović, D., Washburn, S. & Dobrosavljević, V. Novel Metallic Behavior in Two Dimensions. *Phys. Rev. Lett.* **86**, 2625–2628 (2001).

9. Eng, K., Feng, X. G., Popović, D. & Washburn, S. Effects of a parallel magnetic field on the metal-insulator transition in a dilute two-dimensional electron system. *Phys. Rev. Lett.* **88**, 136402 (2002).

10. Pradhan, N. R., McCreary, A., Rhodes, D., Lu, Z., Feng, S., Manousakis, E., Smirnov, D., Namburu, R., Dubey, M., Walker, A. R. H., Terrones, H., Terrones, M., Dobrosavl-

jević, V. & Balicas, L. Metal to insulator quantum-phase transition in few-layered ReS_2. *Nano Lett.* **15**, 8377–8384 (2015).

11. Stanley, L. J., Chuang, H. J., Zhou, Z., Koehler, M., Yan, J., Mandrus, D. & Popović, D. Low-temperature 2D/2D Ohmic contacts in WSe_2 field-effect transistors as a platform for the 2D metal-insulator transition. *ACS Appl. Mater. Interfaces* **13**, 10594–10602 (2021).

12. Sarachik, M. P. "Transport studies in doped semiconductors near the metal-insulator transition" in *The Metal-Nonmetal Transition Revisited: A Tribute to Sir Nevill Mott*, Edwards, P. P. & Rao, C. N., Eds. (Francis and Taylor Ltd.: London, 1995), pp. 79–104.

13. Belitz, D. & Kirkpatrick, T. R. The Anderson-Mott transition. *Rev. Mod. Phys.* **66**, 261–380 (1994).

14. Ando, T., Fowler, A. B. & Stern, F. Electronic properties of two-dimensional systems. *Rev. Mod. Phys.* **54**, 437–672 (1982).

15. Jaroszyński, J. & Popović, D. Aging and memory in a two-dimensional electron system in Si. *Physica B* **404**, 466–469 (2009).

16. Jaroszyński, J., Popović, D. & Klapwijk, T. M. Universal behavior of the resistance noise across the metal-insulator transition in silicon inversion layers. *Phys. Rev. Lett.* **89**, 276401 (2002).

17. Heemskerk, R. & Klapwijk, T. M. Nonlinear resistivity at the metal-insulator transition in a two-dimensional electron gas. *Phys. Rev. B* **58**, R1754–R1757 (1998).

18. Popović, D., Fowler, A. B. & Stiles, P. J. Determination of the threshold voltage in sodium-doped metal-oxide-semiconductor structures. *Phys. Rev. B* **43**, 4495–4498 (1991).
